# Transient Expression of CRISPR/Cas9 Machinery Targeting *TcNPR3* Enhances Defense Response in *Theobroma cacao*

**DOI:** 10.3389/fpls.2018.00268

**Published:** 2018-03-02

**Authors:** Andrew S. Fister, Lena Landherr, Siela N. Maximova, Mark J. Guiltinan

**Affiliations:** ^1^Department of Plant Science, Pennsylvania State University, University Park, PA, United States; ^2^Huck Institutes of the Life Sciences, Pennsylvania State University, University Park, PA, United States

**Keywords:** *Theobroma cacao*, gene editing, defense response, CRISPR/Cas9, *NPR3*, *Phytophthora*, transient transformation

## Abstract

*Theobroma cacao*, the source of cocoa, suffers significant losses to a variety of pathogens resulting in reduced incomes for millions of farmers in developing countries. Development of disease resistant cacao varieties is an essential strategy to combat this threat, but is limited by sources of genetic resistance and the slow generation time of this tropical tree crop. In this study, we present the first application of genome editing technology in cacao, using Agrobacterium-mediated transient transformation to introduce CRISPR/Cas9 components into cacao leaves and cotyledon cells. As a first proof of concept, we targeted the cacao *Non-Expressor of Pathogenesis-Related 3 (TcNPR3)* gene, a suppressor of the defense response. After demonstrating activity of designed single-guide RNAs (sgRNA) *in vitro*, we used *Agrobacterium* to introduce a CRISPR/Cas9 system into leaf tissue, and identified the presence of deletions in 27% of *TcNPR3* copies in the treated tissues. The edited tissue exhibited an increased resistance to infection with the cacao pathogen *Phytophthora tropicalis* and elevated expression of downstream defense genes. Analysis of off-target mutagenesis in sequences similar to sgRNA target sites using high-throughput sequencing did not reveal mutations above background sequencing error rates. These results confirm the function of NPR3 as a repressor of the cacao immune system and demonstrate the application of CRISPR/Cas9 as a powerful functional genomics tool for cacao. Several stably transformed and genome edited somatic embryos were obtained via *Agrobacterium*-mediated transformation, and ongoing work will test the effectiveness of this approach at a whole plant level.

## Introduction

*Theobroma cacao*, the tropical tree which produces cocoa beans, is the centerpiece of the multi-billion dollar chocolate industry and is a vital export for many developing countries. As such, reliable productivity from cacao plants is essential to stabilize the chocolate industry, the economies of producing countries, and the livelihoods of the millions of smallholder cacao farmers (Wood and Lass, 2008).

Each year, infection of cacao by a variety of pathogens severely impacts global production with 20–30% of pods destroyed pre-harvest (Ploetz, 2016). In West Africa, especially severe *Phytophthora* spp. outbreaks can destroy all cacao fruit on a single farm. Because diseases are a persistent problem for cacao, improvement of disease resistance through breeding (Gutiérrez et al., 2016), biocontrol (Ten Hoopen and Krauss, 2016), and biotechnology (Guiltinan and Maximova, 2015; Mondego et al., 2016) approaches are all active areas of research.

The genomics underlying cacao's immune response have been extensively explored in order to better understand the major mechanisms and genes of the cacao defense response. The major classes of cacao pathogen receptor genes and other components of the downstream pathways were first globally described through whole genome sequencing and phylogenetic analysis with model plant systems (Argout et al., 2011). Similarly, a more detailed global description of the pathogenesis-related gene families of cacao was published (Fister et al., 2016a). The cacao *Non-expressor of Pathogenesis-Related 1* (*TcNPR1*) gene, known as the master regulator of the immune system (Cao et al., 1997; Fu and Dong, 2013), was characterized by Shi et al. (2010) and its overexpression resulted in reduction of *Phythophthora* spp. infection in cacao leaf tissue (Fister et al., 2015), Functional genomics analysis of several other cacao candidate defense related genes was also conducted. A class III chitinase gene was demonstrated to reduce infection severity by fungal (Maximova et al., 2006) and oomycete (Fister et al., 2016b) pathogens. NPR3, an NPR1 family member and potential salicylic acid binding protein (Fu et al., 2012; Yan and Dong, 2014), was also characterized in cacao (Shi et al., 2013b). Evidence from Arabidopsis suggests that NPR3 negatively regulates NPR1 activity (Kuai et al., 2015). Similarly, an artificial miRNA-mediated knockdown of *TcNPR3* also resulted in increased resistance to infection (Shi et al., 2013b).

After development of the CRISPR/Cas9 strategy as a tool for targeted genome editing (Jinek et al., 2012), it quickly became a powerful resource for crop improvement (Miao et al., 2013; Schaeffer and Nakata, 2015; Petolino et al., 2016). The system has already been applied to many crops including rice (Miao et al., 2013; Xie and Yang, 2013; Xu et al., 2014; Ikeda et al., 2015; Xie et al., 2015; Wang et al., 2016; Minkenberg et al., 2017), potato (Butler et al., 2015; Andersson et al., 2017), tomato (Brooks et al., 2014; de Toledo Thomazella et al., 2016; Li et al., 2017), wheat (Shan et al., 2013; Wang et al., 2014; Zhang et al., 2016), orange (Jia and Wang, 2014; Peng et al., 2017), soybean (Jacobs et al., 2015; Sun et al., 2015; Du et al., 2016), poplar (Fan et al., 2015; Zhou et al., 2015), cucumber (Chandrasekaran et al., 2016), watermelon (Tian et al., 2017), and cassava (Odipio et al., 2017), and reports of its use in additional species are appearing each month. CRISPR/Cas9 strategies have also been used to knock out and/or edit pathogen effector proteins (Fang and Tyler, 2016), further demonstrating CRISPR/Cas9's versatility for gene characterization in plant pathology.

Based on our previous experience with NPR3's activity as a defense response repressor in cacao (Shi et al., 2013b) we selected this gene as a candidate for development of the CRISPR/Cas9 approach in cacao. Because NPR3 negatively regulates the defense response, we hypothesized that knocking out the gene would result in enhanced resistance in CRISPR/Cas9-treated tissue. To assess this hypothesis without waiting to produce a full cacao tree harboring a *TcNPR3* mutation, we first used a transient transformation approach (Fister et al., 2016b) to introduce the CRISPR/Cas9 components into detached leaf tissue and evaluated the resistance phenotype using an *in vitro* pathogen bioassay.

This strategy resulted in the reproducible mutagenesis of ~27% of *TcNPR3* copies in detached cacao leaves. Moreover, this frequency of mutagenesis enhanced resistance to *P. tropicalis*, a widespread, naturally occurring pathogen of cacao and other crop plants (Alizadeh and Tsao, 1985; Aragaki and Uchida, 2001), resulting in a similar phenotype to that observed from transient overexpression of an artificial miRNA targeting *TcNPR3*. Further, we conducted high-throughput sequencing to search for off-target effects at regions similar in sequence to the single-guide RNA (sgRNA) targets that did not detect off-target mutations. Subsequent transformation of secondary cacao embryos resulted in generation transgenic embryos that appear to show mosaicism for the wild-type and mutant *TcNPR3* copies. As a whole, this study represents the first use of the CRISPR/Cas9 system in *Theobroma cacao*, and embodies an important first step in applying the technique for functional genomics of cacao and toward precision engineering to improve the crop.

## Materials and methods

### Guide design

Using *Geneious* (ver. 9.1.8) and the CRISPR site tool (Drummond et al., 2012; Doench et al., 2014) a table of potential CRISPR target sites was generated for the *T. cacao NPR3* gene sequence using the Criollo cacao genome (v1) (Argout et al., 2011) as the off-target database and a target size of N(20). Potential sgRNAs 60% or higher (Hsu et al., 2013) were further evaluated to ensure they were located in an exon in a domain of interest, did not show allelic variation between Criollo and Scavina 6 genotypes, had 40–80% GC content, and had favorable secondary structure using a method described in Xie et al. (2015) and *RNAfold* Webserver program (http://rna.tbi.univie.ac.at/cgi-bin/RNAWebSuite/RNAfold.cgi) (Hofacker, 2003).

### *In vitro* assay for sgRNA activity

sgRNA templates were PCR-generated using primers Guide Template F and R (see Table S1) containing a T7 promoter region, Cas9 homology region, and the guide sequence being tested. The PCR product was amplified in a 50 μL reaction using Phusion High-Fidelity Polymerase (NEB, Ipswich, MA) and a thermocycler protocol of: 1 min at 98°C, 9 cycles of 30 s 98°C, 15 s at 55°C, 1 min 72°C, and 5 min at 72°C. The products for each sgRNA were cleaned using SpinSmart™ PCR Purification & Gel Extraction Kit (Denville Scientific, Holliston, MA). The templates were used to generate the sgRNAs using HiScribe T7 High Yield RNA Synthesis Kit (NEB) that were quantified using Qubit RNA BR Assay Kit (ThermoFisher Scientific, Waltham, MA). A ~1 kb region centered on each CRISPR target was amplified from cacao DNA (Scavina 6 genotype) using primers NPR3-F1/R1 and NPR3-F2/R2 (Table S1) and these amplicons were also cleaned using SpinSmart columns. For both sgRNA templates, a 30 μl CRISPR/Cas9 activity assay with a 1:20:20 molar ratio of target DNA:Cas9 enzyme (NEB):sgRNA reaction was created and pre-incubated for 10 min at 25°C. The DNA target amplicon was then added and the reaction was incubated for 2h at 37°C. The reaction was terminated by adding 1 μg of Proteinase K (ThermoFisher Scientific), incubated for 10 min at 25°C, and then it was loaded on a 1% agarose gel (IBI Scientific, Peosta, IA) with a 1 KB Plus ladder (ThermoFisher Scientific) stained with GelRed (Biotium, Fremont, CA) for visualization.

### Plasmid construction

To generate the sgRNA-containing region of the vector, forward and reverse primers for each guide were annealed to each other generating double-stranded guide fragments with unique BsaI overhangs. Annealing was performed in 100 uM annealing buffer heated to 95°C and then cooled to 25°C, (Table S1, primers sgRNA1–NPR3 F/R, sgRNA2–NPR3 F/R). They were then ligated into an intermediate vector, pCR3-EF (Staskawicz Lab, UC Berkeley) between the BsaI sites downstream of the AtU6-26 promoter (Waibel and Filipowicz, 1990). The above pCR3-EF-guide plasmids were linearized using Apa1 and SAP enzymes (NEB) to be used as templates for individual amplification reactions (Table S1), where Vec-sgRNA1-F and sgRNA2-U6-R and sgRNA2-F and Vec-U61-R were used to generate an AtU6-26-guide-sgRNA product for each sgRNA. The BsaI overhangs facilitate assembly of these two products with each other and also enable their insertion into the Gateway donor vector via Golden Gate assembly. Amplification was performed in a 50 μL reaction using Phusion Polymerase with the following protocol: 1 min at 98°C, 35 cycles of 20 s at 98°C, 20 s at 55°C, 30 s at 72°C, and 5 min at 72°C. The 641 bp amplicon was loaded on a 1% agarose gel alongside a 1 KB Plus ladder (ThermoFisher Scientific), cut out, and purified using Denville Scientific SpinSmart™ PCR Purification & Gel Extraction Kit. The Golden Gate reaction was set up with pGSh16.0520 (GenBank MF944257, Figure S1), amplified fragments for each guide, 10X T4 buffer, BsaI enzyme and T4 DNA ligase (NEB) for 2 h at 37°C. The resulting product was transformed into 10-beta Competent *E. coli* (NEB). The resulting colonies were purified using Wizard® *Plus* SV Minipreps DNA Purification kit (Promega, Madison, WI), and the sequence was verified via Sanger sequencing. A Gateway LR reaction was performed using Gateway® LR Clonase® enzyme mix following the manufacturer's protocol (ThermoFisher Scientific) and Gateway modified pPZP200 (NovoPro) as the destination vector (ThermoFisher Scientific) resulting in pGSh16.1010 (Figure S2, GenBank MF375491). The product of the LR reaction prior to Golden Gate assembly was also transformed to generate pGSh16.1012 (Figure S3, GenBank MF479729), a control vector lacking sgRNAs.

### Detached leaf transformation and pathogen bioassay

*Theobroma cacao* Scavina 6 trees were grown in a greenhouse at Penn State University under previously described conditions (Swanson et al., 2008). Stage C cacao leaves were collected and transiently transformed using a published protocol (Fister et al., 2016b). Pathogen bioassays were performed on transformed tissues 48 h after *Agrobacterium* infiltration after leaves were screened for successful transformation by monitoring EGFP fluorescence (Fister et al., 2016b). Leaves were inoculated with *Phytophthora tropicalis* isolate Eq 73-73. Infected leaves were photographed 72 h after inoculation as described (Fister et al., 2015). Immediately after photographs were taken, tissue was collected and flash frozen in liquid nitrogen to be used for DNA and RNA extractions. Tissue collection was performed as previously described (Fister et al., 2015). Briefly, leaf discs for DNA extractions were collected using a 1.5 cm cork bore to excise tissue around the lesion (approximately 0.1 g) and are subsequently pooled and treated as one biological replicate. The remaining tissue from the inoculated side of the leaf was collected for RNA extraction (~0.1–0.2 g).

### Nucleic acid extractions

Genomic DNA was extracted from cacao leaves 5 days after *Agrobacterium* infiltration using a modified CTAB protocol (Helliwell et al., 2016), followed by a 0.3 M final concentration sodium acetate/ethanol precipitation. RNA was extracted using Invitrogen Plant RNA Reagent (Invitrogen, Carlsbad, CA) with minor modifications of the recommended protocols. These include: 1 ml of plant RNA reagent was added to each ground tissue sample, 0.2 ml of 5 M NaCl were added to samples prior to chloroform extraction, and all centrifugations were performed at 14,000 rpm.

### Detection of mutagenized DNA

The 2 kb *TcNPR3* target region was amplified via PCR with Phusion Polymerase (NEB). Each 50 μl reaction contained 10 μl 5x HF Phusion buffer, 0.5 μl Phusion enzyme, 0.5 μM final concentration of primers (NPR3-F1 and NPR3-R2, Table S1), 200 μM final concentration of dNTPs, and ~20 ng template DNA. The thermocycler protocol was 98°C for 30 s, 30 cycles of 30 s 98°C denaturation, 30 s of 60°C annealing, and 75 s of 72°C extension, followed by a final 5 min extension. Samples were subsequently A-tailed to allow ligation into a T-overhang cloning vector by adding 1 unit of Taq for 30 min at 72°C.

PCR products were quantified using a Qubit Fluorometer (ThermoFisher), and ~100 ng of each PCR product was loaded per lane onto a 1% agarose gel stained with 0.02% GelRed (Biotium). To verify that the amplified fragments were the correct sequences, upper and lower bands from CRISPR/Cas9-treated samples were excised from the gel, purified using a GeneClean II kit (MPBio, Santa Ana, CA), ligated into pGEM T cloning vector (Promega, Madison, WI), transformed into 10-beta competent *E. coli* (NEB), and miniprepped as described above. Extracted DNA was Sanger sequenced using the T7 and SP6 primers.

Quantitative PCR was performed using an ABI 7300 StepOnePlus Real-Time PCR system (Applied Biosystems) to quantify the proportion of *TcNPR3* copies cut by Cas9. Two primer sets (Table S2) were designed to amplify fragments of *TcNPR3*: NPR3 del F/R amplify a fragment within the deletion region (amplicon 1) and NPR3 out F/R amplify a region of exon 1 (outside of the deletion region) (amplicon 2). Reactions were performed in 10 μL volumes using SYBR Premix Ex Taq reagents (Clontech, Mountain View, CA) with 10 ng of template DNA and final primer concentrations of 0.4 μM. CT values were used to calculate the ratio of amplicon 1/amplicon 2. Eight biological replicates were included, and each reaction was performed in technical duplicate.

### Quantitative PCR for pathogen bioassay evaluation

RNA samples were treated with DNase as described (Zhang et al., 2015). RNA was quantified using a Qubit 3 Fluorometer. 500 ng of RNA was used for cDNA synthesis as described (Zhang et al., 2015). All quantitative PCR was performed as described above using an ABI 7300 StepOnePlus Real-Time PCR system (Applied Biosystems, Foster City, CA) and SYBR Premix Ex Taq reagents (Clontech, Mountain View, CA). DNA qPCR for pathogen quantification was performed as described in Wang et al. (2011), using the *P. tropicalis Actin* and *T. cacao Actin7* genes as targets (Table S2). qRT-PCR of PR genes, *TcNPR1*, and *TcNPR3* was performed using previously described primers (Zhang et al., 2015), and used the reference gene *TcTubulin1* as an endogenous control (Table S2).

### Statistical methods

The transient transformation and subsequent *P. tropicalis* infection assay were repeated three times as described above. Data was analyzed using a mixed model in which treatment was a fixed effect and experimental repetition (block) was a random effect. The model calculated significance of the treatment effect (*p* < 0.05) for lesion size and DNA ratio, and pairwise *t*-tests were used to assign treatments to significance groups for the measurements. The block effect from repeated experiments was not significant (Wald *p* = ~0.6). For analysis of PR gene expression, transcript abundance was calculated relative to the reference gene *TcTubulin1* using the ΔΔCt method (Livak and Schmittgen, 2001), and fold induction was calculated using REST (Pfaffl et al., 2002).

### Analysis of off-target mutagenesis

Off-target sites were identified using Geneious and those with highest off-target scores were selected. Geneious generated five off-target sites for each guide. Using the Criollo cacao genome database (Argout et al., 2011), primers with Illumina adapter sequences were designed to amplify 100–200 bp regions centered on each off-target site. These primers were used to amplify the off-target sites from DNA samples derived from ten leaves transformed with pGSh16.1010 (CRISPR/Cas9) and ten leaves transformed with pGSh16.1012 (vector control). PCR was performed using Phusion enzyme and the reaction setup described above with a similar thermocycling protocol, with a 52°C annealing temp for 8 of the 9 primer sets (Table S3), a 49°C annealing temp for the sgRNA2 OT5, and a 15 s extension time in each cycle. 5 μl of the PCR products were electrophoresed on 1% agarose gel stained with GelRed (Biotium) to confirm amplification of a single band. PCR products were cleaned using SpinSmart PCR cleanup columns (Denville), and Sanger sequenced to confirm presence of the off-target site in the amplicon. We were never able to amplify off-target five for sgRNA 1, and off-target five for sgRNA 2 had more differences between its sequence and that of the sgRNA, so its off-target activity score is not known, but is likely quite low. These problems were likely the result of differences between the genotype used for transformation (Scavina 6) and that of the cacao reference genome (Criollo).

Amplicons from the nine successfully amplified off-target sites for each of the 20 samples were mixed into 20 equimolar pools corresponding to the original leaf samples. Pools were submitted to Penn State's Genomics Core Facility and were used to prepare TruSeq DNA Nano Libraries (Illumina, San Diego, CA). Libraries were sequenced on a 150 nt single read Miseq (Illumina) run.

Sequences of the ~100–200 bp regions centered around off-target sites were concatenated into a “pseudo-genome” which was used as a mapping reference for the Miseq dataset using bwa mem (version: 0.7.15) (Li, 2013). Variants were called using Freebayes (v1.0.2) variant calling program (Garrison and Marth, 2012) and were visualized in IGV (Thorvaldsdóttir et al., 2013). Truncated alignments were removed and only alignments starting within 50 base pairs of the selected sgRNA-like sequences and aligning to at least half their length were considered.

To select random off-target sites for analysis, a python script was written to randomly select 100 sites with an average of >10,000x coverage across the ten samples.

### Stable transformation and detection of mutation in cacao somatic embryo

Secondary PSU Sca6 somatic embryo cotyledons were transformed as previously described (Maximova et al., 2003). Beginning 4 weeks after culture initiation, embryos were viewed every 4 weeks to monitor development of transgenic tissue and select transgenic embryos as previously described (Maximova et al., 2006). The transgenic embryos were cultured and multiplied as in (Li et al., 1998). DNA was isolated from a 2 mm diameter circle of cotyledon tissue collected from CRISPR/Cas9 transgenic (pGSh16.1010), transgenic control (pGSh16.1012), and non-transgenic PSU Sca6 embryos using Phire Plant Direct PCR Master Mix (Thermo Scientific), using the dilution and storage method for plant leaves. A collection control was performed by dipping the collection tool in the dilution buffer without any tissue to ensure no cross contamination between samples. The manufacturer's protocol was used to set up a 20 μl reaction using NPR3- and NPR3-2R1F, Table S1, 60°C annealing temperature and 35 cycles to amplify the *TcNPR3* target region. For size comparison, *TcNPR3* target region was amplified from DNA extracted from transiently transformed leaves as described above. The PCR reactions were electrophoresed and visualized as described above.

The lower band, representing the *TcNPR3* copy harboring the deletion, was again purified from the gel, cloned into pGEM T cloning vector, and 10 clones were Sanger sequenced. Sequence data were aligned to the *TcNPR3* reference gene using Geneious.

The nine predicted off-target sites were also amplified from the CRISPR/Cas9-treated embryo DNA using Phire Plant Direct PCR Master Mix. These amplicons were also cloned into pGEM T cloning vector, and a minimum of five clones of each were sequenced. Again, cloned sequences were compared to the Criollo genome's reference sequence in Geneious.

All experiments received institutional biosafety committee approval (PSU IBC #47447, “Molecular Biology of Cacao”).

## Results

### sgRNA design and *in vitro* efficacy

**Figure 1:**
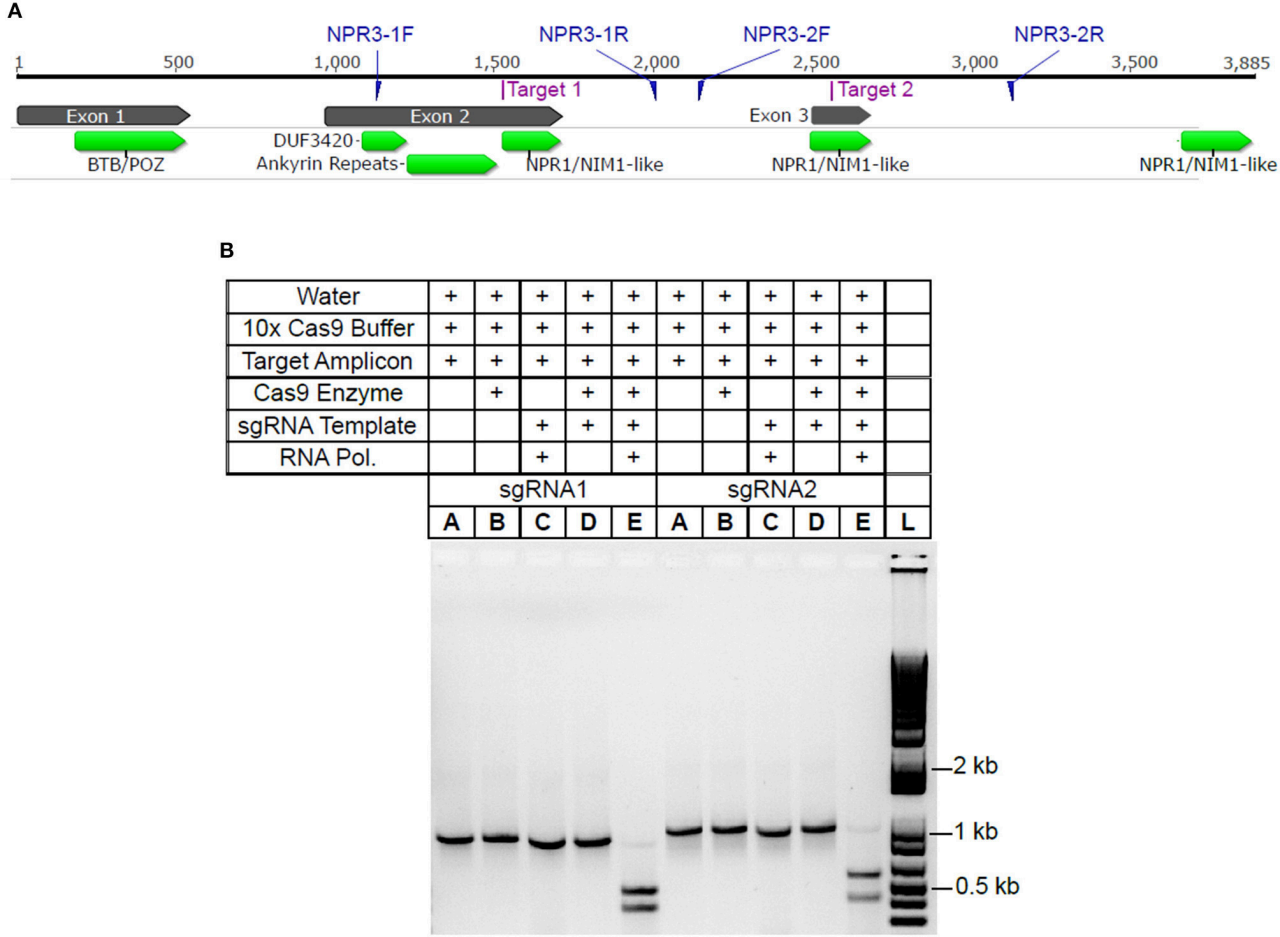
*TcNPR3* gene model and demonstration of sgRNA activity *in vitro* **(A)**. *TcNPR3* gene model based on the Criollo genome sequence. Numbers above the gene model indicate length in bp, starting at base 1 of the ATG start codon. Target sites for selected sgRNAs and primer binding sites for amplification of sgRNA target amplicons are indicated. Functional domains were predicted using CDSearch (Marchler-Bauer et al., 2015). **(B)** Agarose gel electrophoresis demonstrating activity of sgRNAs in *in vitro* assay of sgRNAs 1 and 2, corresponding to Target 1 and Target 2 in 1A. Table above gel indicates reagents and templates added to each reaction and loaded into each lane. sgRNA template and RNA polymerase indicates presence/absence in sgRNA synthesis reaction preceding Cas9 activity assay. Lane L was loaded with 1 KB Plus Ladder (NEB).

Using the CRISPR site finder toolset in Geneious v9 (Kearse et al., 2012), we identified potential sgRNA target sites within the *TcNPR3* gene (Criollo gene ID Tc06_g011480). Two sgRNA sequences were selected, each of which targeted protospacer adjacent motifs (PAM) in an exon of *TcNPR3* (Figure 1A). They also had target scores <0.6 and had favorable predicted secondary structure (Hofacker, 2003). Activity of each sgRNA was then assessed *in vitro* by mixing a ~1 kb PCR amplicon containing the respective sgRNA target with synthesized sgRNA and recombinant Cas9 endonuclease. Reactions were electrophoresed and target amplicon cleavage was only observed in reactions containing both Cas9 protein and an sgRNA (Figure 1B).

### Binary vector construction and *in vivo* mutagenesis

**Figure 2:**
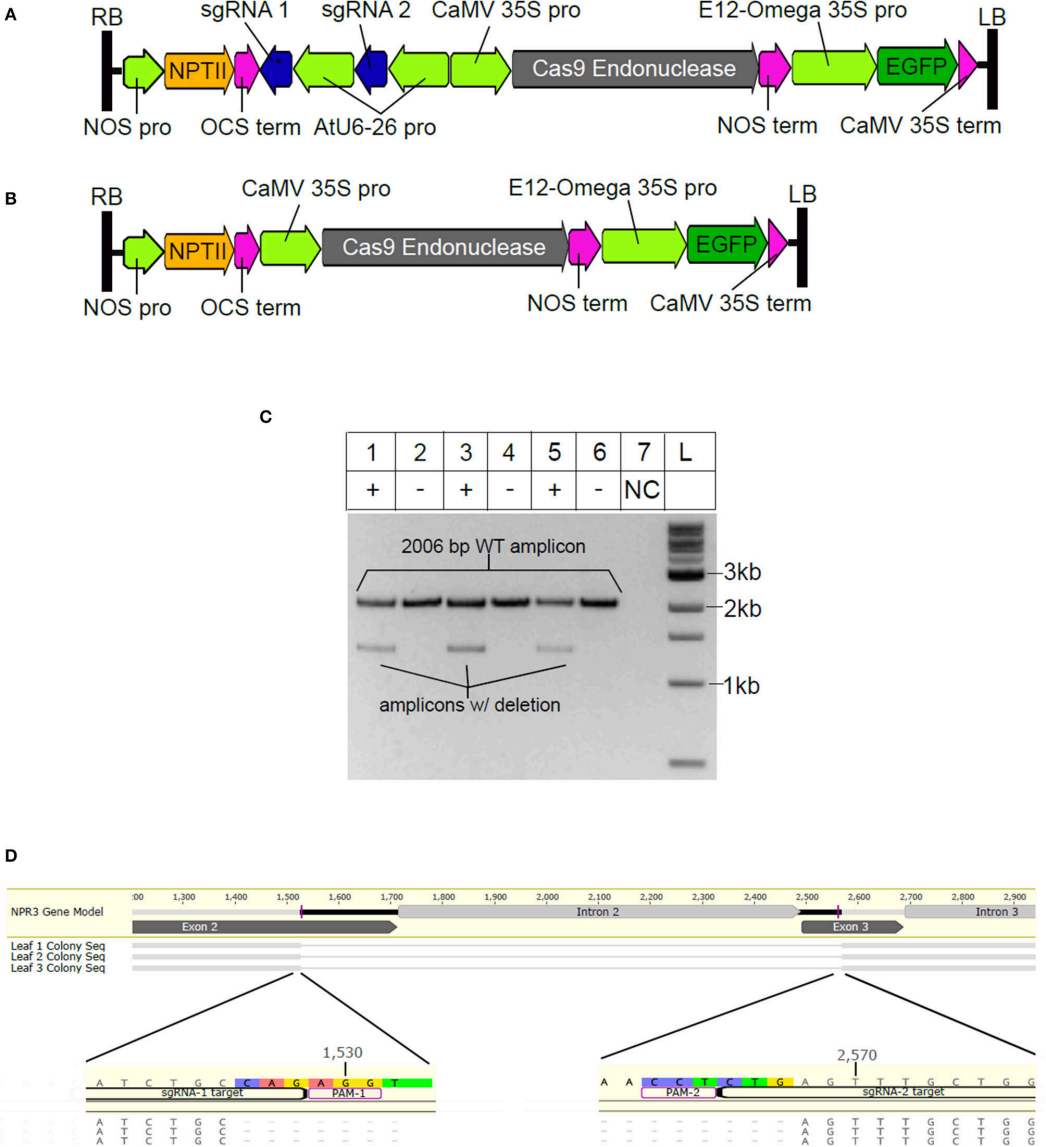
Vector design and demonstration of sgRNA activity *in vivo*. **(A)** T-DNA region of vector pGSh16.1010. RB and LB indicate right and left borders, **(B)** T-DNA region of vector pGSh16.1012, a vector control lacking sgRNAs. **(C)** Agarose gel electrophoresis of *TcNPR3* target region. For this analysis, three different leaves were cut into two sections and the two sections of each leaf were transformed with either pGSh16.1010 (+) or pGSh16.1012 (-). DNA from each pair of samples was extracted and used as a PCR template in separate reactions (lanes 1 and 2, lanes 3 and 4, lanes 5 and 6). Lane 7 (labeled NC) contains a control PCR reaction (no DNA template). Lane L contains 500 ng of 1 kb ladder (NEB). **(D)** Alignment of sequences cloned from lower bands extracted from gel in (C). NPR3 Gene Model is the reference sequence from the Criollo genome database. Labels Leaf 1, 2, and 3 correspond to DNA samples used as template for reactions in lanes 1, 3, and 5 of **(C)**.

Having demonstrated that the selected sgRNAs were able to efficiently direct targeted Cas9 endonuclease activity *in vitro*, we designed an *Agrobacterium* T-DNA binary vector to introduce CRISPR/Cas9 machinery into cacao cells. Two binary vectors were constructed, pGSh16.1010 (Figure 2A, Figure S2, GenBank: MF375491) which contains cassettes for Cas9 and the NPR3 targeted sgRNA expression driven by the Arabidopsis AtU6-26 promoter within its T-DNA region, and pGSh16.1012 (Figure 2B, Figure S3, GenBank: MF479729), which contains a Cas9 expression cassette but lacking the sgRNA cassette.

Stage C cacao leaves were transiently transformed using these vectors via *Agrobacterium*-mediated transformation as previously described (Fister et al., 2016b). After 2 days of co-cultivation, the leaves were also subjected to pathogen assay as previously described (Fister et al., 2016b) and after another 3 days, DNA was extracted from leaves transformed with each vector and used for PCR amplification of a target region within *TcNPR3* to assess if cleavage of the target site had occurred. We hypothesized that in some proportion of cells, sgRNAs would mediate cleavage at both of their target sites leading to a ~1,044 bp deletion. Therefore, in samples transformed with the CRISPR/Cas9 machinery we predicted amplification of a 2,006 bp wild-type PCR product and a 962 bp truncated product resulting from the deletion. As expected, in all three CRISPR/Cas9-treated replicate samples, we observed the wild type amplicon and a smaller band, albeit running more slowly than expected (Figure 2C). Each of the smaller fragments were excised from the gel, purified, cloned and Sanger sequenced, confirming that they were the 962 bp predicted deletion product (Figure 2D). It is likely that the repetitive nature of the *TcNPR3* coding sequence contributes to slightly slower electrophoretic mobility relative to the molecular markers. The deletion also results in the creation of a premature stop codon 45 bp downstream of the point where exons two and three fuse, which is predicted to lead to translational termination within the second NPR1/NIM1-like domain.

### Quantification of *in vivo* mutagenesis

**Figure 3:**
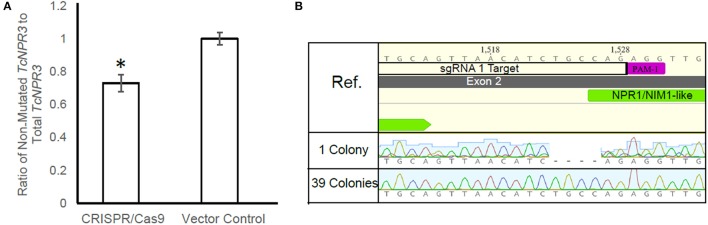
Quantification of gene editing frequency. **(A)** qPCR measurement of the ratio of *TcNPR3* lacking a deletion to total *TcNPR3*. Bars represent means calculated from eight replicates, error bars are standard error from eight replicates. Asterisk denotes *t*-test *p* < 0.05. **(B)** Alignment of electropherograms derived from cloning and sequencing the upper band of *TcNPR*3 target region PCR product from a CRISPR/Cas9-transformed cacao leaf. Ref. is *TcNPR3* sequence from the Criollo genome browser (Argout et al., 2011).

The *Agrobacterium*-mediated transient gene delivery system we utilized for this study was previously demonstrated to result in gene expression in only a fraction of the cacao leaf cells (Fister et al., 2016b). Therefore, we quantified the proportion of cells in cacao leaves edited by CRISPR/Cas9 machinery by using qPCR to measure the proportion of *TcNPR3* copies cut by CRISPR/Cas9. Eight additional leaf samples were transiently transformed with pGSh16.1010 and pGSh16.1012 and DNA was again extracted and used as a template for amplification of a region within the deletion caused when both guides mediate cleavage and a region of *TcNPR3* outside of the deletion. We calculated a 27% reduction in the ratio of the uncut to total *TcNPR3* in CRISPR/Cas9 samples relative to vector control treated samples (*t*-test *p* < 0.05; Figure 3A).

To assess the proportion of *TcNPR3* copies harboring a mutation at a single guide site, we electrophoresed an amplification of *TcNPR3* as shown in Figure 2C, then purified the upper band which was then cloned and DNA was isolated and sequenced from 40 transformed *E. coli* colonies. One colony contained a 4 bp Cas9-induced deletion two base pairs upstream from sgRNA1 target's PAM site (Figure 3B). None of the colonies had mutations in the sgRNA2 target area. This provided evidence that in a small proportion of events, a single sgRNA directed DNA cleavage and was followed by imperfect DNA repair resulting in the small deletion.

### Effect of *TcNPR3* mutagenesis on pathogen resistance and downstream gene activation

**Figure 4:**
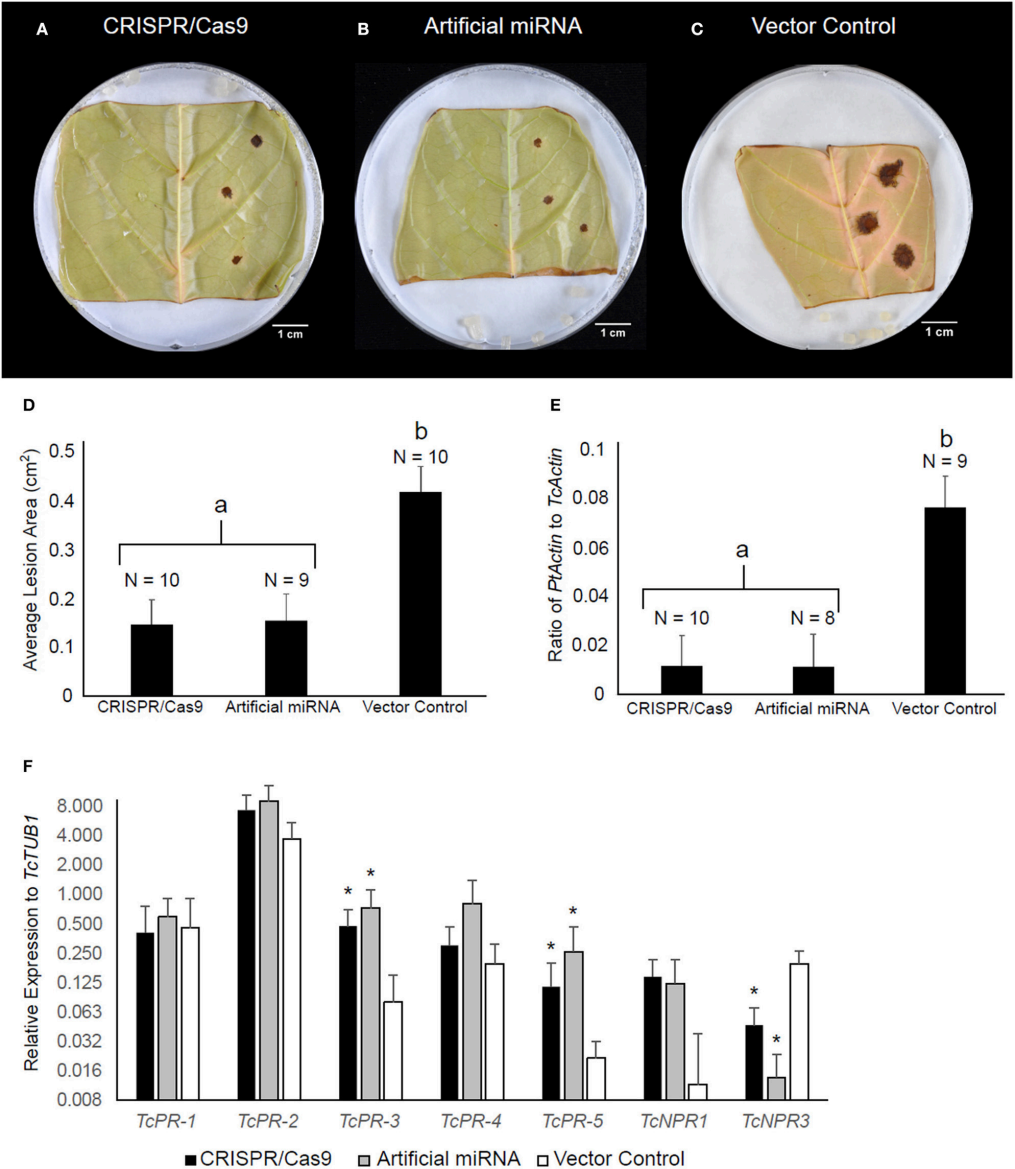
*TcNPR3* mutagenesis by CRISPR/Cas9 increases resistance to *Phytophthora tropicalis* in detached cacao leaf. Representative photographs of *P. tropicalis* inoculated leaves transformed with **(A)** pGSh16.1010 (*TcNPR3* CRISPR/Cas9 system) **(B)** pGS12.0225 (*TcNPR3* artificial miRNA), and **(C)** pGSh16.1012 (vector control). **(D)** Bar graph of lesion size analysis. Bars represent mean lesion size and error bars represent standard error. N represents number of samples across three experimental repetitions. Letter labels indicate statistically significantly differing treatments (*t*-test *p* < 0.05). **(E)** Bar graph of DNA ratio analysis. Bars represent mean ratio of *P. tropicalis* actin to *T. cacao* actin and error bars represent standard error. N represents number of samples across three experimental repetitions. Letter labels indicate statistically significantly differing treatments (*t*-test *p* < 0.05). **(F)** Relative expression of defense-related genes after transient transformation and *P. tropicalis* inoculation. Bars represent mean expression value across nine samples from three experimental repetitions, and error bars represent standard error. Asterisks indicate statistically significant increase in expression relative to vector control treatment detected in REST (Pfaffl et al., 2002).

NPR3 was previously shown in Arabidopsis and cacao to act as a repressor of NPR1-dependent defense gene activation and pathogen resistance (Fu et al., 2012; Shi et al., 2013a,b). Therefore, we predicted that the deletion within the *TcNPR3* gene would result in increased disease resistance in edited cacao tissue. To test this prediction, transiently transformed tissues were also subjected to a pathogen bioassay as previously described (Fister et al., 2016b). As a positive control, we also transiently transformed leaves with pGS12.0225 (Shi et al., 2013b), a binary vector containing an artificial miRNA targeting *TcNPR3*, previously shown to reduce infection symptoms from *Phytophthora* inoculation (Shi et al., 2013b). As predicted, 72 h after inoculation, CRISPR/Cas9-and miRNA-treated tissues exhibited similarly reduced lesion sizes relative to the vector control (Figures 4A–D) in three replicated experiments (*p* < 0.05). We also conducted qPCR to calculate the ratio of pathogen to cacao DNA as a proxy for pathogen replication, and found significant reduction (*p* < 0.05) in pathogen DNA in the CRISPR/Cas9- and miRNA-treated samples compared to the vector control (Figure 4E).

We also analyzed expression of defense-related genes in these transformed and infected leaves. Transcript abundances of *TcNPR3, TcNPR1*, and five pathogenesis-related (PR) genes were measured by qRT-PCR (Figure 4F). We noted a trend of elevated gene expression for five genes (*PR-2, PR-3, PR-4, PR-5*, and *TcNPR1*) after transformation with CRISPR/Cas9 and artificial miRNA-treated samples relative to vector control-treated samples. Elevated expression was statistically significant in two of these cases: PR-3 and PR-5 showed 5.5- and 5.9-fold higher expression (*p* < 0.05) respectively in CRISPR/Cas9-treated samples, while in artificial miRNA-treated samples they were increased 7.2- and 11-fold (*p* < 0.01). *TcNPR3* expression was also significantly affected by CRISPR/Cas9 and artificial miRNA treatment. In the CRISPR/Cas9 treated leaves, its expression was reduced to approximately one fifth of that in the vector control treated leaves, and expression was reduced to one eleventh that of the control by the artificial miRNA (*p* < 0.05). Therefore, while transient introduction of CRISPR/Cas9 only appears to mutate ~27% of *TcNPR3* copies, we observed a more dramatic effect on the gene's expression throughout the treated leaves.

### Analysis of off-target mutations

**Figure 5:**
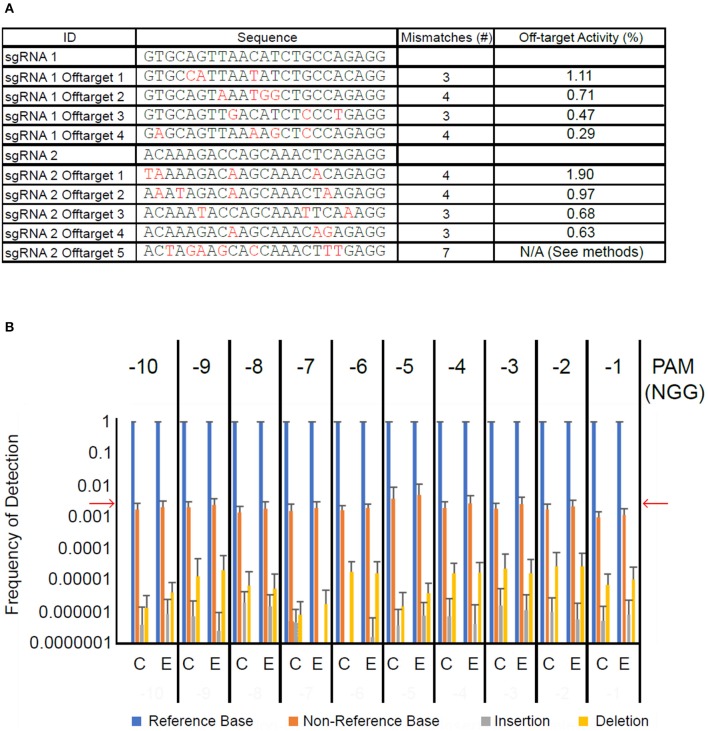
High-throughput sequencing of off-target sites does not detect elevated mutation frequency. **(A)** Table of sgRNA sequences and nine selected off-target sequences. Base pair differences between off-target sites and their corresponding guide are in red. The last column indicates the predicted off-target activity score calculated in Geneious. **(B)** Bar graph summarizing results of Miseq experiment. Framing above graph indicates position (number of bases upstream) relative to PAM site. Bars represent average frequency of each possible sequencing result across the nine off-target sites and ten DNA pooled amplicon samples each for vector control transformed leaf tissue (C) and CRISPR/Cas9-transformed leaf tissue (E). Error bars represent standard deviation. Red arrows indicate approximate Miseq substitution error rate (Ross et al., 2013).

Next, we sought to determine whether transient introduction of CRISPR/Cas9 machinery led to off-target mutagenesis of cacao DNA. Using the *Geneious* CRISPR site tool, we identified the off-target sites in the Criollo cacao genome predicted to be most likely off-targets of our sgRNAs (Argout et al., 2011). Those we selected differed from the sgRNA target sites at three to seven positions (Figure 5A). We first amplified 100–200 bp regions surrounding each of the nine off target sites from 10 samples treated with CRISPR/Cas9 vector and 10 treated with control vector. These amplicons were pooled such that the nine amplicons from each sample were mixed together at equimolar concentrations. We next used high-throughput sequencing to detect mutations induced at these nine off-target sites. Each amplicon within each pool had extreme sequencing coverage ranging from ~7,000x to ~200,000x. Because CRISPR/Cas9 mutagenesis generally occurs a few bases upstream of the PAM site, we counted the proportion of reads in the alignment showing a base matching the reference sequence, a base other than that in the reference sequence, an insertion, or a deletion at these sites. Measurement of these frequencies in the vector control-treated tissue served as a background level of sequencing error, and the reference base was called in >99% of reads (Figure 5B), consistent with previous measurements of Miseq error rates (Ross et al., 2013). Non-reference bases were detected at a frequency of ~0.01–~0.001. Insertions and deletions were detected on average in less than one in 10,000 reads. We detected the same base-call frequencies in CRISPR/Cas9-treated tissue. Frequencies were consistently low across for all nine amplicons (Figures S4A–I), and were also consistent for a randomly selected set of bases within the amplicons but not in the 10 bp upstream from the PAM (Figure S4J). These results suggest that if off-target mutagenesis is occurring at the predicted sites, it is at a rate below the inherent error rate of the Miseq system.

### Generation of stably transgenic *TcNPR3* mutant embryos

**Figure 6:**
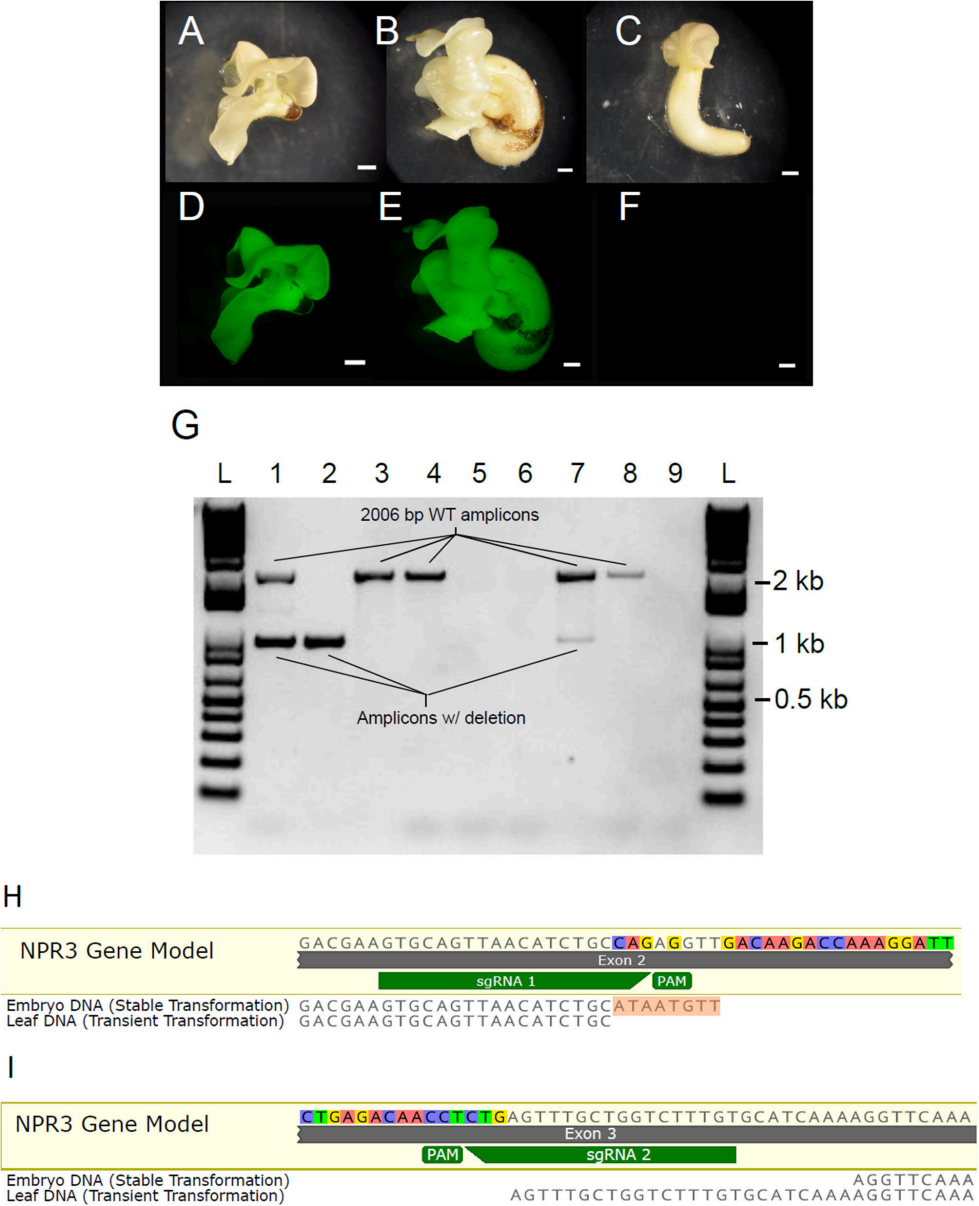
Generation of somatic embryos containing *TcNPR3* deletion. Photographs of somatic embryos derived from **(A)** pGSh16.1010 transformed tissue, **(B)** pGSh16.1012 transformed tissue, **(C)** untransformed tissue. Scale bars represent 1 mm. Photographs of EGFP fluorescence from the same embryos, **(D)** pGSh16.1010 transformed tissue, **(E)** pGSh16.1012 transformed tissue, **(F)** untransformed tissue. **(G)** Agarose gel electrophoresis of *TcNPR3* target region from transformed embryos. L: 1 KB Plus Ladder (NEB). Lanes 1 and 2: Amplification of *TcNPR3* from two excised cotyledon pieces from a pGSh16.1010 transformed embryo. Lane 3: Amplification of *TcNPR3* from an excised piece of pGSh16.1012 transformed embryo. Lane 4: Amplification of *TcNPR3* from untransformed tissue. Lane 5: Attempted amplification from collection control. Lane 6: No template negative control for PCR from embryos. Lane 7: Amplification of *TcNPR3* target region from DNA extracted from pGSh16.1010 transiently transformed leaf. Lane 8: Amplification of *TcNPR3* target region from pGSh16.1012 transiently transformed leaf. Lane 9: No template negative control for PCR from leaves. **(H)** Alignment of mutant *TcNPR3* allele amplified from embryo to sgRNA 1 and **(I)** sgRNA 2 target sites. 8 bp insertion in embryo allele is highlighted in orange. Mutant allele detected in leaf transient assays also aligned for comparison. NPR3 Gene Model is the reference sequence from the Criollo genome browser.

Having demonstrated the efficacy and specificity of the CRISPR/Cas9 system using the transient assay, we next transformed cotyledons of secondary embryos in order to recover plants containing the partial *TcNPR3* deletion. Embryos were recovered from tissue transformed with pGSh16.1010 and pGSh16.1012 (Figures 6A–F). Two embryos were recovered from pGSh16.1010 transformations, and both of these develop more slowly than those recovered from vector control or non-transgenic tissue. One of these died before it could be sampled for DNA extraction. DNA was isolated from the remaining embryo and was used as template for amplification of the *TcNPR3* target region (Figure 6G). PCR products from DNA extracted from different cotyledons of the embryo were electrophoresed in Lanes 1 and 2 as shown in Figure 6A. Lane 1 shows fragments representing both the wild type and mutant versions of the gene, while lane 2 only contains the mutant *TcNPR3* band, suggesting that either the embryo is a mosaic of wild type and mutant cells and/or containing some cells that are heterozygous for the deletion. Bands amplified from DNA extracted from embryos matched the sizes of fragments detected in DNA from transiently transformed leaves.

To assess the *TcNPR3* allele sequence in the mutant embryo, we sequenced the lower band and found that the embryo contained a different mutant allele than what was detected in leaf transient transformations (Figures 6H,I). At the sgRNA 1 predicted cut site, we found that the deletion again began 3 bp upstream from the PAM. However, the embryo contained an 8 bp insertion (shown in Figure 6H). The sgRNA 2 cut site in the embryo began 28 bp upstream from the PAM, creating a 25 bp longer deletion than that created in leaf transient transformations (Figure 6I).

We also amplified, cloned, and sequenced the nine predicted off-target sites from DNA extracted from the mutant embryo. Sequencing five clones for each site, we did not detect any off-target mutation (data not shown). However, given that the sgRNAs and Cas9 protein are stably integrated into the genome, mutations could occur later in embryo or plant development.

## Discussion

Genome editing techniques stand to revolutionize plant biotechnology. Long generation time crops, including cacao, may benefit most from deployment of these techniques, as precisely engineered plants can be created quickly and at low cost, without the labor or space required for a large mutation screen experiment. Besides the tremendous potential for crop improvement, genome editing techniques also offer a new means of assessing gene function. Genome editing techniques have already proven useful in improving plant defense response via editing of resistance genes (Wang et al., 2014), inhibiting transcription of susceptibility genes (Li et al., 2012; Peng et al., 2017), knockout of transcription factors (Wang et al., 2016, 2017), and cleaving viral DNA (Green and Hu, 2017; Khatodia et al., 2017).

In this study we used a CRISPR/Cas9 genome editing approach for the first time in cacao, and targeted the *TcNPR3* gene, which was previously shown to be an important regulator of cacao's defense response (Shi et al., 2013b). We first used a transient transformation approach to show the efficacy of CRISPR/Cas9 machinery in cacao leaf tissue, and subsequently transformed secondary cotyledons to recover stably transgenic, *TcNPR3* mutant cacao embryos that can be grown into plants.

Generally, this transient transformation system proved to be a relatively fast means of showing sgRNA efficacy *in vivo* and can be applied in the future as a first screen before proceeding toward generating gene-edited plants. Additionally, we were able to detect an increased resistance phenotype from the *TcNPR3* mutation after mutagenizing on average 27% of *TcNPR3* copies in the transiently transformed leaf tissue. The ability to detect CRISPR induced mutation phenotypes within a few days provides us with a powerful functional genomics tool and a way to assess the potential phenotype of any given genomic editing design prior recovery of mutagenized plants. Our results indicate that in the case of major pathway regulators like NPR3, mutation of only a fraction of copies in a leaf may be sufficient to trigger downstream processes and result in a strong phenotypic change. We hypothesize that cells in which *TcNPR3* is mutated by transient transformation signal to nearby cells, activating their defense responses. Induction of the defense pathway should lead to the downregulation of *TcNPR3* throughout the leaf and induction of PR genes and other defense components, and our gene expression measurements were consistent with this prediction. The systemic acquired resistance pathway includes a complex network of signaling including NPR1 turnover, ROS burst, and epigenetic modification of defense genes (Fu et al., 2012; Fu and Dong, 2013; Mauch-Mani et al., 2017). Future work will investigate the molecular mechanisms connected to *TcNPR3* knockout, and this work will be aided by further analysis of mature cacao trees harboring this *TcNPR3* deletion. Also, the decrease in pathogen susceptibility in edited tissue was comparable to that in leaf tissue treated with an artificial miRNA previously used by our group Shi et al. (2013b). Collectively these results suggest that once mature, our *TcNPR3* mutant embryos will exhibit an enhanced defense phenotype.

Off-target mutagenesis is a concern and potential limitation for deployment of CRISPR/Cas9 for crop improvement (Wolt et al., 2016). In other plants and other organisms, studies have reported low frequencies of mutagenesis at loci similar to sgRNA targets (Jacobs et al., 2015; Peng et al., 2017). First, we used a Miseq approach to evaluate off-target effects in transiently transformed tissue, and we were not able to detect off-target mutations above the Miseq sequencing error rate (approximately 0.001). However, considering that Xie and Yang reported detection of off-target mutagenesis after transient CRISPR/Cas9 expression in rice protoplasts (Xie and Yang, 2013), deep sequencing on an individual cell basis is likely required to definitively understand the scope of off-target events. We also cloned and sequenced the off-target sites from the *TcNPR3* mutant embryo we generated, and did not detect any off-target effects. Given that these can occur at low frequency and may continue to occur given the stable integration of the sgRNAs and Cas9, sequencing the genome of a *TcNPR3* mutant tree once it reaches maturity may yield a different result.

After transforming secondary cotyledons, we noted embryos forming from CRISPR/Cas9-treated tissue grew very slowly. This may be due to metabolic drag resulting from *TcNPR3* deletion, which could result in a constitutively activated defense response. Previous work showed that NPR3 mutant Arabidopsis had lower seed weight and shorter root length, indicating some growth-related effects from loss of NPR3 function (Shi et al., 2013a). After extracting DNA from CRISPR/Cas9 embryos, we found that both the wild type and mutant *TcNPR3* genes could be amplified from one of the samples. This could be the result of mosaicism, in which embryos developed from a mix of wild type cells and some containing the mutation. Another possibility is that the embryo developed from a single cell heterozygous for insertion of the T-DNA. Over time, expression of the sgRNAs and Cas9 in the developing embryos could continue to increase the proportion of the mutated *TcNPR3* version in this tissue. In this case, it is possible that the embryo or plant will eventually become homozygous for the mutation. The *TcNPR3* mutant embryo we generated can be multiplied through secondary embryogenesis (Maximova et al., 2002), which will also be evaluated to determine the proportion of cells containing the deletion.

Ultimately this set of experiments displayed the utility of our transient leaf transformation protocol as a means of easily introducing CRISPR/Cas9 components into cacao for precise genome editing and shows that tissue culture can be used to recover embryos recovered after mutagenesis with CRISPR/Cas9. Future analyses of other target genes will determine whether genome editing in detached leaves is a robust approach for assessing the effect of mutating ~27% of copies of other genes. Regardless, we demonstrated the efficacy of the CRISPR/Cas9 system in cacao and will continue to explore its applications for studying cacao biology and genetic improvement.

## Genbank accession numbers

pGSh16.1010 (MF375491), pGSh16.1012 (MF479729), pGSh16.0520 (MF944257), Figures S1, S2, S4.

## Author contributions

AF designed experiments and performed transient transformations, nucleic acid extractions, quantitative PCR, data analysis, and drafted and edited the manuscript. LL designed and evaluated sgRNAs, performed the *in vitro* Cas9 assay, led vector construction and validation, transformed and validated somatic embryos, and contributed to drafting the manuscript. SM and MG developed the project, oversaw experimental progress, and contributed to drafting and editing the manuscript.

### Conflict of interest statement

The authors declare that the research was conducted in the absence of any commercial or financial relationships that could be construed as a potential conflict of interest.
