# Supplementary figures and images for: Transient Expression of CRISPR/Cas9 Machinery Targeting TcNPR3 Enhances Defense Response in Theobroma cacao
Source: Front Plant Sci. 2018 Mar 2;9:268. doi: 10.3389/fpls.2018.00268 (PMC5841092; doi:10.3389/fpls.2018.00268)

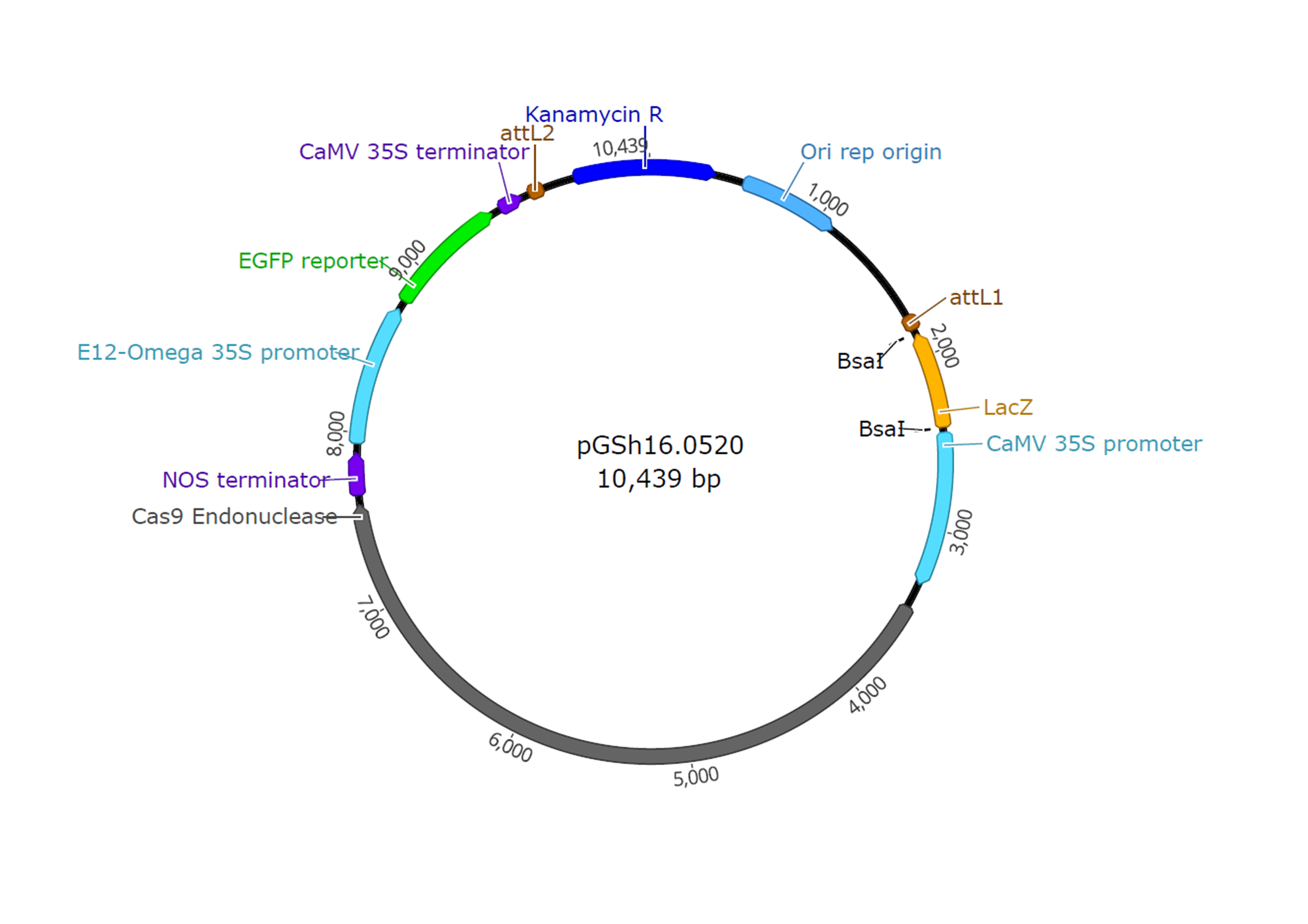

Supplement: Supplementary file 1 [file Image1.TIF]

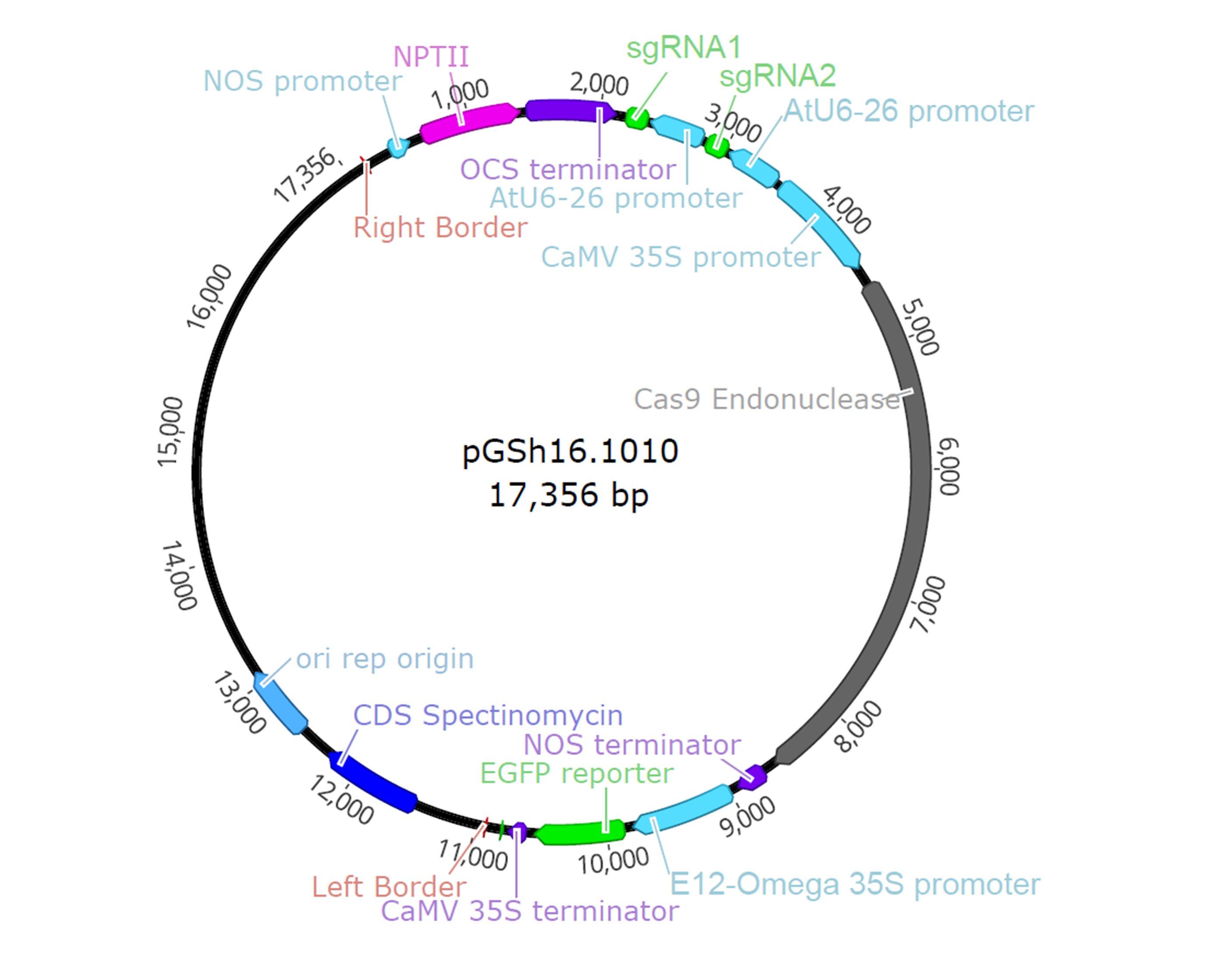

Supplement: Supplementary file 2 [file Image2.TIF]

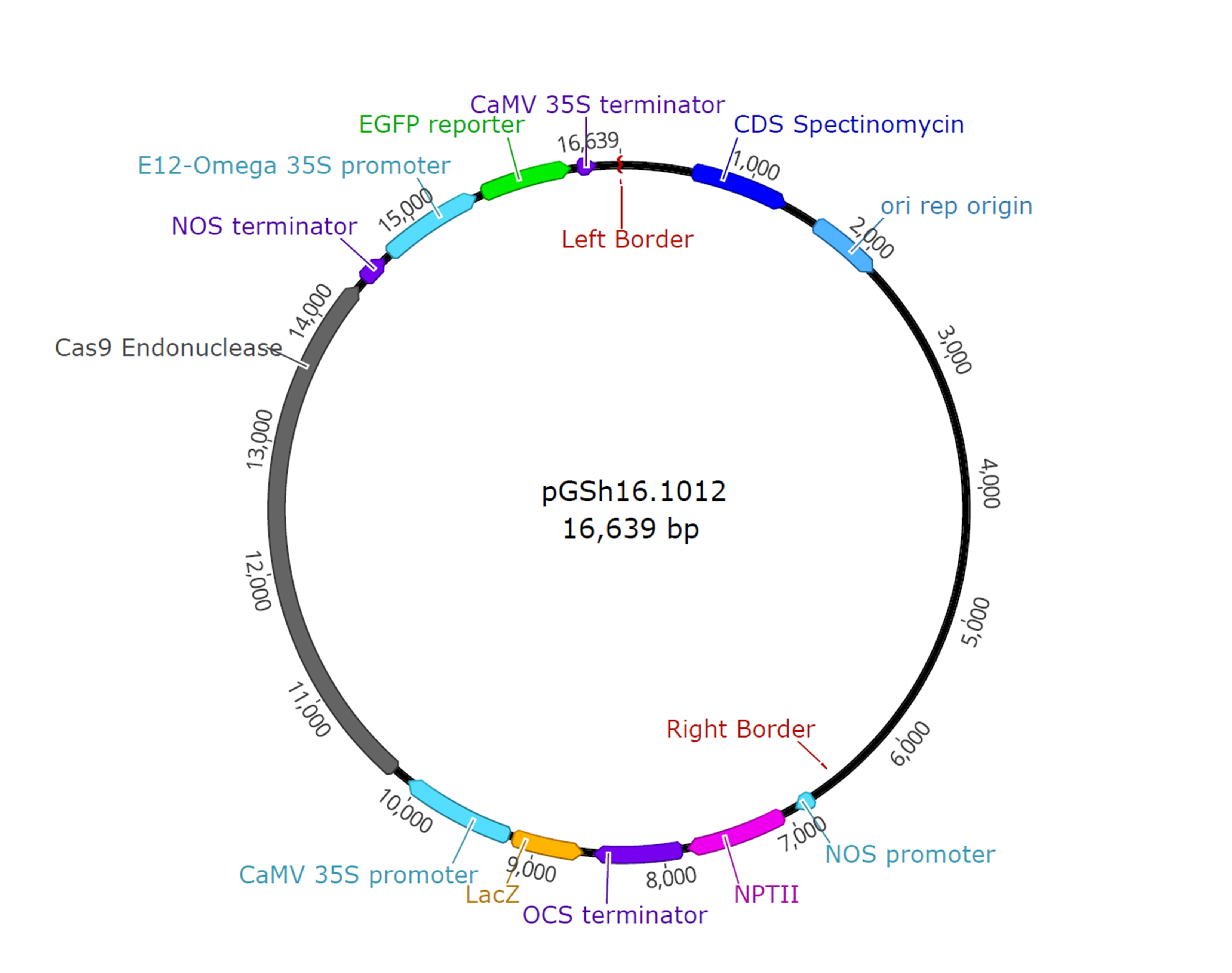

Supplement: Supplementary file 3 [file Image3.TIF]

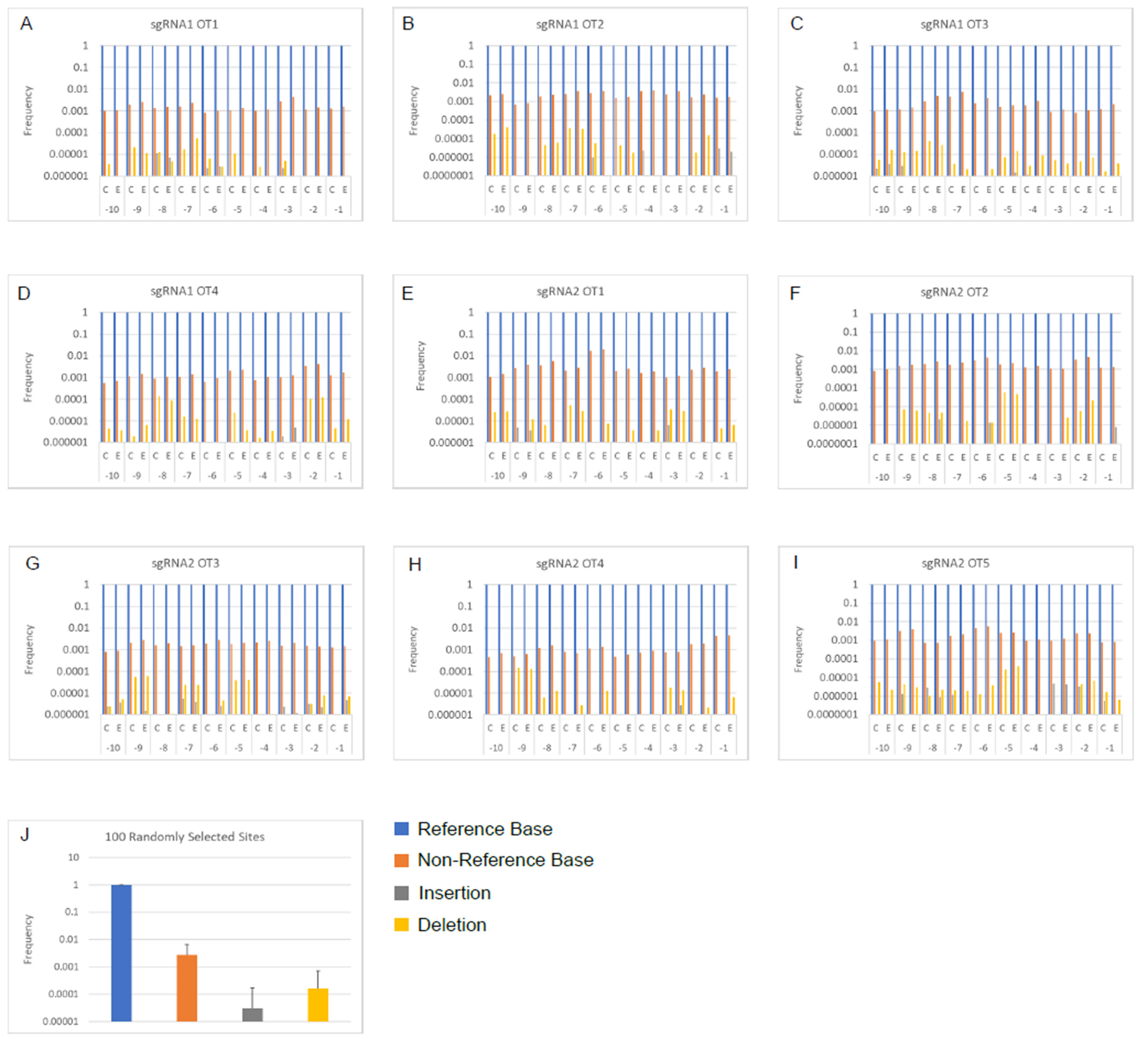

Supplement: Supplementary file 4 [file Image4.TIF]
