# Supplementary material for: Transient Expression of CRISPR/Cas9 Machinery Targeting TcNPR3 Enhances Defense Response in Theobroma cacao
Source: Front Plant Sci. 2018 Mar 2;9:268. doi: 10.3389/fpls.2018.00268 (PMC5841092; doi:10.3389/fpls.2018.00268)
Supplement: Supplementary file 5 [file Table1.DOCX]

**Supplemental Files**

**Fig. S1** – Full vector diagram for pGSh16.0520.

**Fig. S2** – Full vector diagram for pGSh16.1010.

**Fig. S3** – Full vector diagram for pGSh16.1012.

**Fig. S4** – Off-target mutation analysis for 9 selected off-target sites and random non-target bases. **A-I)** Base detection frequencies for off-target sites. Bars represent mean frequency of detection from ten DNA samples derived from tissue transformed with vector control (C) and CRISPR/Cas9 vector (E). Framing below graphs indicates position relative to PAM. **J)** Frequencies detected at 100 randomly selected non-sgRNA-like bases in control vector treated samples. Bars represent mean frequency of detection averaged across the 100 bases. Error bars are standard deviation calculated from means from the ten samples and across the 100 bases.

**Table S1** – Primers for vector construction and *in vitro* sgRNA activity assay. For simplicity, codes were assigned to primers used for amplification of TcNPR3 regions. These codes are shown in parentheses.

| **Oligo Name** | **Sequence** |
| --- | --- |
| Guide Template F  (bold T7 promoter sequence) | **TAATACGACTCACTATAGGG**-(20 bp guide sequence)-*gtttAagagctaTGCTGgaa* |
| Guide Template R  (bold Cas9 homology) | **aaaagcaccgactcggtgccactttttcaa**  **gttgataacggactagccttattt**AaacttgctaTGCTGtttcCAGCAtagctctTaaac |
| sgRNA1-NPR3 | GTGCAGTTAACATCTGCCAG |
| sgRNA2-NPR3 | ACAAAGACCAGCAAACTCAG |
| NPR3 assay sgRNA1-F (NPR3-F1) | GCGGAGAGTATTCGATTGCT |
| NPR3 assay sgRNA1-R (NPR3-R1) | GCTGACTAACACTTCAAAATTCCC |
| NPR3 assay sgRNA2-F (NPR3-F2) | GTCATGCTGGAACTGTAGCC |
| NPR3 assay sgRNA2-R (NPR3-R2) | GAGAAGATGTGTCCAACTGTCC |
| sgRNA1-NPR3 F | attgGTGCAGTTAACATCTGCCAG |
| sgRNA1-NPR3 R | aaacCTGGCAGATGTTAACTGCAC |
| sgRNA2-NPR3 F | attgACAAAGACCAGCAAACTCAG |
| sgRNA2-NPR3 R | aaacCTGAGTTTGCTGGTCTTTGT |
| Vec-sgRNA1-F(aaac) | GGATCCGGTCTCA(aaac)CCG CGGATATCCA CTAGACAGAAGTTT |
| sgRNA2-F(tact) | GGATCCGGTCTCA(tact)CCG CGGATATCCA CTAGACAGAAGTTT |
| Vec-U61-R(attg): | ACTCGAGGTCTCG(attg)AAG CTTTCGTTGA ACAACGGAAACTCGACTTG |
| sgRNA2-U6-R(agta) | ACTCGAGGTCTCG(agta)AAG CTTTCGTTGA ACAACGGAAACTCGACTTG |

**Table S2** – qPCR and RT-qPCR primers. Gene IDs correspond to the Criollo cacao genome database V1 (Argout et al., 2011).

| **Primer Name** | **Criollo V1 Gene ID** | **Sequence** |
| --- | --- | --- |
| NPR3 del F | Tc06g011480 | ACAGAGCAAGGAAAGGAAACA |
| NPR3 del R | Tc06g011480 | CAGCCAACGTATGGGAAGTAA |
| NPR3 out F | Tc06g011480 | ATGGGTCTTGTGAGAAAAAGG |
| NPR3 out R | Tc06g011480 | CTCAGCTTCCCAGTGTACAAATA |
| PcActin qPCR F | N/A | GACAACGGCTCCGGTATGTGCAAGG |
| PcActin qPCR R | N/A | GTCAGCACACCACGCTTGGACTG |
| Actin7 Realtime F | Tc01g010900 | AGCTGAGAGATTCCGTTGTCCAGA |
| Actin7 Realtime R | Tc01g010900 | CCCACATCAACCAGACTTTGAGTTC |
| TcPR1 Realtime F | Tc02g002410 | CCTCAATGCTCACAACACGGCTC |
| TcPR1 Realtime R | Tc02g002410 | CGCTGCTCATTGCAAGGTTCTC |
| TcPR2 Realtime F | Tc04g029300 | CCTTGCTAACCCTTCCAATGCACAG |
| TcPR2 Realtime R | Tc04g029300 | CCAAGGCAGGCAAAACAAATTGAGC |
| TcPR3 Realtime F | Tc04g018160 | GCACAACCGGTGACCTTACTACC |
| TcPR3 Realtime R | Tc04g018160 | CTGTTCCCTTATAAAGCAATATCCCCATG |
| TcPR4 Realtime F | Tc05g027210 | GCATAGTCCACAGTAAGGTGACCTTG |
| TcPR4 Realtime R | Tc05g027210 | CGCTTCCAATGTGAGAGCTACTTACC |
| TcPR5 Realtime F | Tc03g026990 | ATTAGATGCACGGCAGATATCATAG |
| TcPR5 Realtime R | Tc03g026990 | CAGAACACAACCCTGTAGTTAGTCC |
| TcNPR1 Realtime F | Tc09g007660 |  |
| TcNPR1 Realtime F | Tc09g007660 |  |
| TcNPR3 Realtime F | Tc06g011480 |  |
| TcNPR3 Realtime F | Tc06g011480 |  |
| TcTub1 Realtime F | Tc06g000360 | GGAGGAGTCTCTATAAGCTTGCAGTTGG |
| TcTub1 Realtime F | Tc06g000360 | ACATAAGCATAGCCAGCTAGAGCCAG |

**Table S3** – Primers for generation of off-target amplicons for Miseq run.

| **Primer Name** | **Sequence (Illumina Adapter in bold)** |
| --- | --- |
| sgRNA1 OT1 MiseqF | **TCGTCGGCAGCGTCAGATGTGTATAAGAGACAG**CCTCAAGTGGTAATGTTGTT |
| sgRNA1 OT1 MiseqR | **GTCTCGTGGGCTCGGAGATGTGTATAAGAGACAG**CTTAAACAGGCCGAATTC |
| sgRNA1 OT2 MiseqF | **TCGTCGGCAGCGTCAGATGTGTATAAGAGACAG**CAGAAAATCACAAAGGCTATG |
| sgRNA1 OT2 MiseqR | **GTCTCGTGGGCTCGGAGATGTGTATAAGAGACAG**AGAAATTCAACTGAAATTTGC |
| sgRNA1 OT3 MiseqF | **TCGTCGGCAGCGTCAGATGTGTATAAGAGACAG**TTACTGGAGAGGATATTAGGATATC |
| sgRNA1 OT3 MiseqR | **GTCTCGTGGGCTCGGAGATGTGTATAAGAGACAG**TGTAGTCCTGCTACTTTCTGC |
| sgRNA1 OT4 MiseqF | **TCGTCGGCAGCGTCAGATGTGTATAAGAGACAG**ACATATCGTTGGATGGC |
| sgRNA1 OT4 MiseqR | **GTCTCGTGGGCTCGGAGATGTGTATAAGAGACAG**CCAAGAATGAGTCAGGTG |
| sgRNA2 OT1 MiseqF | **TCGTCGGCAGCGTCAGATGTGTATAAGAGACAG**ATATACGCTTACAAATTGCAA |
| sgRNA2 OT1 MiseqR | **GTCTCGTGGGCTCGGAGATGTGTATAAGAGACAG**ACAGGACTTATCTTAATCATAATATG |
| sgRNA2 OT2 MiseqF | **TCGTCGGCAGCGTCAGATGTGTATAAGAGACAG**AGATGGGGCATGTGAAC |
| sgRNA2 OT2 MiseqR | **GTCTCGTGGGCTCGGAGATGTGTATAAGAGACAG**TTATTTAGCCTTATATCAGAAATATT |
| sgRNA2 OT3 MiseqF | **TCGTCGGCAGCGTCAGATGTGTATAAGAGACAG**CTCCTCCAAACTACGATGTTG |
| sgRNA2 OT3 MiseqR | **GTCTCGTGGGCTCGGAGATGTGTATAAGAGACAG**ACAAGGTTCTGCACCTTCTC |
| sgRNA2 OT4 MiseqF | **TCGTCGGCAGCGTCAGATGTGTATAAGAGACAG**TAGGATCTTCCTACATGGAC |
| sgRNA2 OT4 MiseqR | **GTCTCGTGGGCTCGGAGATGTGTATAAGAGACAG**ACCAGAATTGAAATTTACAGAA |
| sgRNA2 OT5 MiseqF | **TCGTCGGCAGCGTCAGATGTGTATAAGAGACAG**TCTCTCATTTCAACTCTCTTCCC |
| sgRNA2 OT5 MiseqR | **GTCTCGTGGGCTCGGAGATGTGTATAAGAGACAG**ATTACAAAACCCAGATAAAAACACC |
